# Optimizing water and nitrogen management strategies to improve their use efficiency, eggplant yield and fruit quality

**DOI:** 10.3389/fpls.2023.1211122

**Published:** 2023-09-11

**Authors:** Chenli Zhou, Hengjia Zhang, Shouchao Yu, Xietian Chen, Fuqiang Li, Yong Wang, Yingying Wang, Lintao Liu

**Affiliations:** ^1^ College of Agronomy and Agricultural Engineering, Liaocheng University, Liaocheng, China; ^2^ College of Water Conservancy and Hydropower Engineering, Gansu Agricultural University, Lanzhou, China

**Keywords:** eggplant, water and nitrogen management, yield, quality, water and nitrogen use efficiency

## Abstract

With improvement in living standards, consumer preferences for vegetables are changing from quantity- to quality-oriented. Water and nitrogen supply, as two major determinants of vegetable crop yield and quality, can be optimally managed to improve the yield and quality. To evaluate the response in yield, fruit quality, and water and nitrogen utilization of eggplant to different water and nitrogen management strategies, a 2-year (2021 and 2022) field trial under mulched drip irrigation was conducted. The growth period was divided into seedling, flowering and fruit set, fruit development, and fruit ripening stages. Three irrigation levels were applied during the flowering and fruit set stage: W0, adequate water supply (70%–80% of field water capacity, FC); W1, mild water deficit (60%–70% FC); and W2, moderate water deficit (50%–60% FC). In addition, three nitrogen application rates were applied: N1, low nitrogen level (215 kg ha^−1^); N2, medium nitrogen level (270 kg ha^−1^); and N3, high nitrogen level (325 kg ha^−1^). The irrigation and nitrogen rates were applied in all combinations (i.e., nine treatments in total). Adequate water supply throughout the reproductive period in combination with no nitrogen application served as the control (CK). The yield of the W1N2 treatment was significantly increased by 32.62% and 35.06% in 2021 and 2022, respectively, compared with that of the CK. Fruit soluble protein, soluble solids, and vitamin C contents were significantly higher under W1 than W2. Fruit quality was significantly higher under the N2 rate compared with the other nitrogen rates. The W1N2 treatment showed the highest water productivity, with a significant increase of 11.27%–37.84% (2021) and 14.71%–42.48% (2022) compared with that under the other treatments. Based on the average water-deficit degree and nitrogen application rate, W0 and N1 had the highest partial factor productivity of nitrogen. Assessment of the results using the TOPSIS (technique for order preference by similarity to an ideal solution) method indicated that mild water deficit in combination with the medium nitrogen application rate (W1N2) was the optimal water and nitrogen management strategy for cultivated eggplant. The present findings contribute novel insights into the sustainable cultivation of eggplant in an oasis arid environment.

## 1 Introduction

Eggplant (*Solanum melongena* L.) is the third-most important crop in the Solanaceae in terms of the harvested area and yield (Dou et al., 2022). Eggplant is a popular vegetable crop that is grown and consumed worldwide (Toppino et al., 2020; Zhou et al., 2021), and produces fruit of high nutritional value. The fruit contain essential nutrients for human health, such as vitamins, minerals, and amino acids, as well as bioactive compounds (Nisha et al., 2009; Karimi et al., 2021). Eggplant is among the most important vegetables grown in summer and autumn throughout China (Dou et al., 2022) and elsewhere in the world (Olak et al., 2018). Ongoing population growth requires sufficient food production to meet the increasing global food demand (Lahoz et al., 2016). In addition, with rapid economic development and improvement of living standards, consumer preferences in food have changed from quantity- to quality-oriented (Li et al., 2021). The eggplant yield and quality of fruit are not only influenced by genotype but are also strongly associated with environmental factors, such as climate change, soil water content, and nutrient availability (Rosales et al., 2011). Water and nitrogen fertilizer are the most critical and easily managed environmental determinants in agricultural production (Li et al., 2021). Therefore, the establishment of an effective water and nitrogen management strategy is fundamental to increase local eggplant yields and fruit quality as well as improve crop water productivity (WP) and nitrogen utilization.

In the interior of west-central China, where rainfall and available water resources are severely limited and evapotranspiration is extremely high (Meng et al., 2013), irrigation is a decisive driver of crop development and agricultural sustainability (Duan et al., 2004). Thus, it is essential to alleviate the water scarcity by establishing novel irrigation strategies to decrease agricultural water consumption and increase resource utilization (Chen et al., 2018). The function of irrigation is to timely provide a certain volume of water required for normal physiological activities of crops (Zhang et al., 2022). In practice, farmers often achieve higher yields through excessive irrigation (Xiao et al., 2023). However, irrigation that exceeds the plant demand or field water capacity (FC) can lead to an imbalance between vegetative and reproductive growth (Reddy, 2016), thereby reducing yield, WP, and fruit quality (Rostamza et al., 2011; Matteau et al., 2022). In contrast, appropriate deficit irrigation helps to achieve a balance between plant vegetative and reproductive growth (Han et al., 2023), reducing plant height while maintaining crop yields, improving fruit quality, and maximizing WP (Wu et al., 2021). Furthermore, deficit irrigation influences crop physiology, and soil nutrient mineralization and absorption, and severe deficit irrigation constrains crop growth, ultimately leading to a dual decrease in yield and quality (Wang et al., 2012).

Nitrogen is among the essential nutrients required for plant growth. An appropriate nitrogen application rate is important to promote the development of plant roots, and enhance the ability to absorb soil moisture and nitrogen, and thereby increase crop yield and fruit quality (Jiang et al., 2017). However, some agricultural activities (such as cultivation and harvesting) can lead to a reduction in the total nitrogen content of surface soils (Urioste et al., 2006). When the nitrogen supply to the soil is insufficient, plant growth is inhibited, and ultimately the yield declines (Lambers et al., 2008). To avoid soil nutrient deficiencies and to achieve higher yields and greater economic returns, farmers often apply large amounts of fertilizers to fields (Chen et al., 2004). In vegetable production, the nitrogen fertilizer input often exceeds the amount needed for crop growth by several-fold, motivated by its greater yield-enhancing effect than other nutrients and the lower input costs (Ju et al., 2006). Nevertheless, excessive nitrogen application can result in accumulation of residual nitrogen or ammonia volatilization in the soil (Fernandez et al., 2020), and there is a risk of nitrate leaching during heavy precipitation (Wei et al., 2009), which can lead to groundwater contamination and groundwater eutrophication (Gong et al., 2011), reduce crop yield, quality, and resource utilization (Zhang et al., 2011; Lang et al., 2018), and increase production costs (Sun et al., 2013). Under drought stress, an appropriate nitrogen application can enhance the drought resistance of plants and simultaneously reduce the negative influences of the stress on crop growth (Waraich et al., 2011). Therefore, it is of vital importance for sustainable agricultural development to optimize nitrogen management strategies to suppress nutrient and nitrate leaching, and improve crop yield, nitrogen utilization, and fruit quality.

The growth, fruit character, yield and quality of continuous cropping eggplant under the irrigation halving treatment were better than those under other treatments (Miao et al., 2019). Rational nitrogen fertilizer rates not only significantly increase eggplant yield and improve the nutritional quality of the fruit, but also increase nutrient accumulation in the plant (He, 2020). Previous research has predominantly concentrated on irrigation and nitrogen management to enhance eggplant yield and quality. Fewer studies have examined the combined effects of water and nitrogen interactions on eggplant. However, in practice, irrigation and nitrogen application need to be managed simultaneously. Whether optimal irrigation and nitrogen application strategies can simultaneously enhance eggplant yield, fruit quality, and resource utilization is currently unclear (Mwinuka et al., 2021). In the present research, we hypothesized that it is possible to establish an optimal level of deficit irrigation and nitrogen application rate that best balances crop evapotranspiration, yield, water and nitrogen utilization, and fruit quality of eggplant. Therefore, the aims of this study were 1) to evaluate the combined effects of different deficit irrigation levels and nitrogen application rates on eggplant evapotranspiration, yield, water productivity, nitrogen utilization, and fruit quality, and 2) to determine the optimal deficit irrigation level and nitrogen application rate using the technique for order preference by similarity to an ideal solution (TOPSIS) for multi-objective optimization to trade-off yield and fruit quality.

## 2 Materials and methods

### 2.1 Experimental site

**Figure 1:**
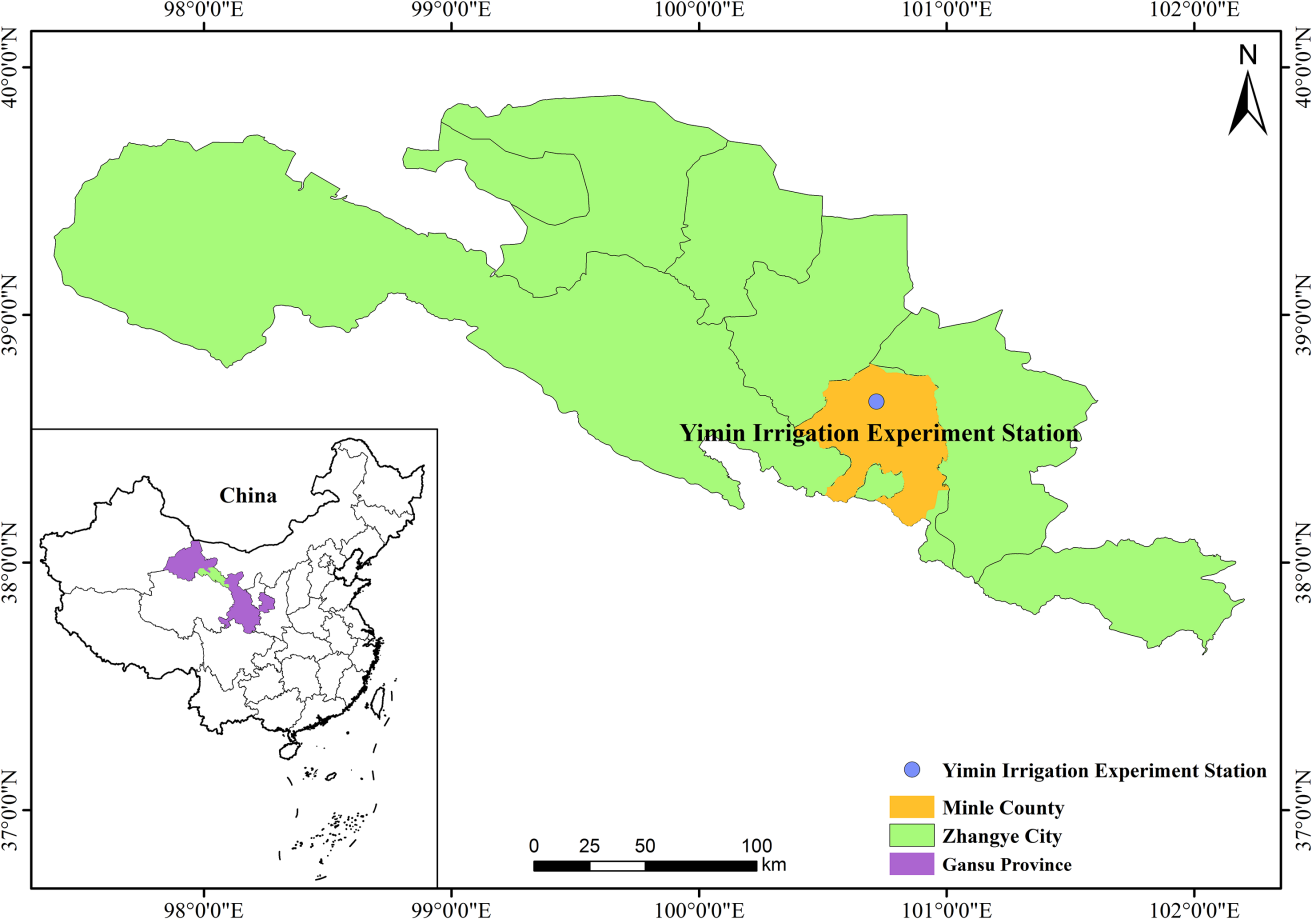
Location of the study site in Minle County, Gansu Province, China.

The experiment was conducted in 2021 and 2022 at the Yimin Irrigation Experiment Station (38°39′′N, 100°43′′E), Minle County, Zhangye City, Gansu Province, China (**Figure 1**). The region has a typical continental desert steppe climate. The long-term average annual rainfall ranges from 183 to 345 mm, the average annual evaporation is 1638 mm, and the average annual temperature is 7.6°C. The annual sunshine duration is 2932 h, the desiccation index is 5.85, and the frost-free period is approximately 150 days. The agricultural soil type is a light loam. The soil bulk density is approximately 1.46 g cm^−3^ (0–60 cm soil layer). The FC of the 0–100 cm soil layer is approximately 24% (gravimetrically measured). The water table in the study area is located at a depth of more than 20 m.

**Figure 2:**
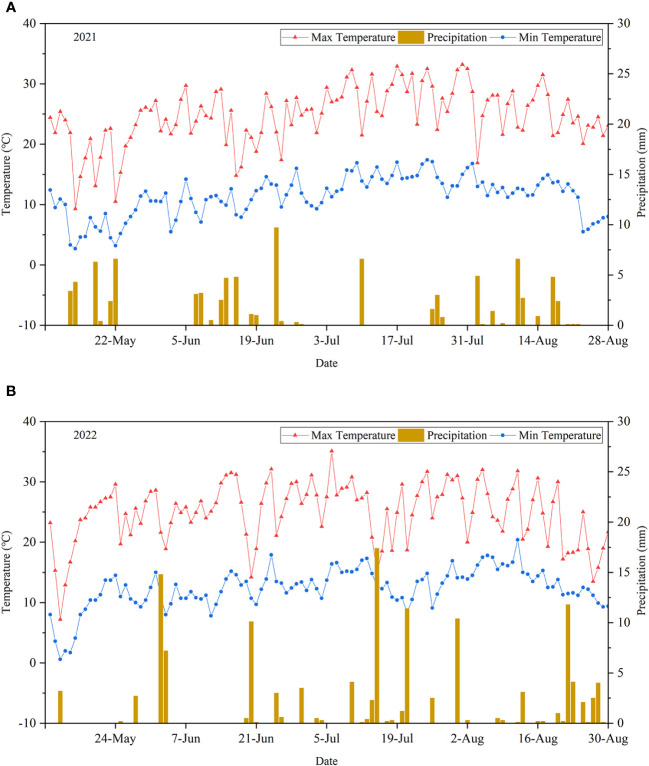
Precipitation, daily maximum temperature, and daily minimum temperature in the study area from May to August in 2021 **(A)** and 2022 **(B)**.

During the two growing seasons, meteorological data were obtained from automatic weather stations installed at the experimental station and from the Minle County Meteorological Bureau. **Figure 2** shows the precipitation, and daily maximum and minimum temperatures in the study area from May to August in 2021 and 2022. In 2021 and 2022, the average daily maximum temperature was 25.3°C and 26.2°C, while the average daily minimum temperature was 6.6°C and 3.4°C, respectively. The total precipitation in 2021 and 2022 was 91.1 mm and 127.6 mm, respectively. A high-rainfall event of more than 15 mm occurred on July 15, 2022.

### 2.2 Experimental design and management

In this study, a complete block design was adopted with nine combinations of water and nitrogen treatments and one control. The field trial was designed as a two-factor test, with irrigation level as the main factor and nitrogen application rate as the secondary factor. Based on plant characteristics, the growth period of eggplant was divided into four stages, comprising seedling (May 9–June 8, 2021; May 11–June 3, 2022), flowering and fruit set (June 4–July 5, 2021; June 4–July 5, 2022), fruit development (July 6–August 2, 2021; July 6–August 2, 2022), and fruit ripening (August 3–August 28, 2021; August 3–August 30, 2022). Three soil moisture levels were applied: W0, adequate water supply (soil moisture maintained at 70%–80% of FC); W1, mild water deficit (soil moisture maintained at 60%–70% of FC); and W2, moderate water deficit (soil moisture maintained at 50%–60% of FC). In addition, three nitrogen application rates were tested: N1, low nitrogen level (215 kg urea ha^−1^); N2, medium nitrogen level (270 kg urea ha^−1^); and N3, high nitrogen level (325 kg urea ha^−1^). The mild and moderate water deficit applications were applied at the flowering and fruit set stage, whereas the adequate water supply was provided at the other growth stages. The adequate water supply (W0) and no nitrogen application (N0) combination was applied as the control (CK). Three replications were performed for all treatments, comprising a total of 30 plots, each of 12 m^2^ (2 m × 6 m). The experimental treatments are summarized in **Table 1**.

**Table 1:** Summary of the combinations of irrigation and nitrogen application rate treatments applied in the study.

Treatment	Water deficit control					Nitrogen control	
	Soil moisture level	Seedling	Flowering and fruit set	Fruit development	Fruit ripening	Nitrogen application level	Nitrogen application rate (kg ha^−1^)
W_0_ N_1_	W_0_	70%–80%	70%–80%	70%–80%	70%–80%	N_1_	215
W_0_ N_2_		70%–80%	70%–80%	70%–80%	70%–80%	N_2_	270
W_0_ N_3_		70%–80%	70%–80%	70%–80%	70%–80%	N_3_	325
W_1_ N_1_	W_1_	70%–80%	60%–70%	70%–80%	70%–80%	N_1_	215
W_1_ N_2_		70%–80%	60%–70%	70%–80%	70%–80%	N_2_	270
W_1_ N_3_		70%–80%	60%–70%	70%–80%	70%–80%	N_3_	325
W_2_ N_1_	W_2_	70%–80%	50%–60%	70%–80%	70%–80%	N_1_	215
W_2_ N_2_		70%–80%	50%–60%	70%–80%	70%–80%	N_2_	270
W_2_ N_3_		70%–80%	50%–60%	70%–80%	70%–80%	N_3_	325
CK	W_0_	70%–80%	70%–80%	70%–80%	70%–80%	N_0_	0

For the water deficit levels, the percentages indicate the amount of water supplied relative to the field water capacity.

**Figure 3:**
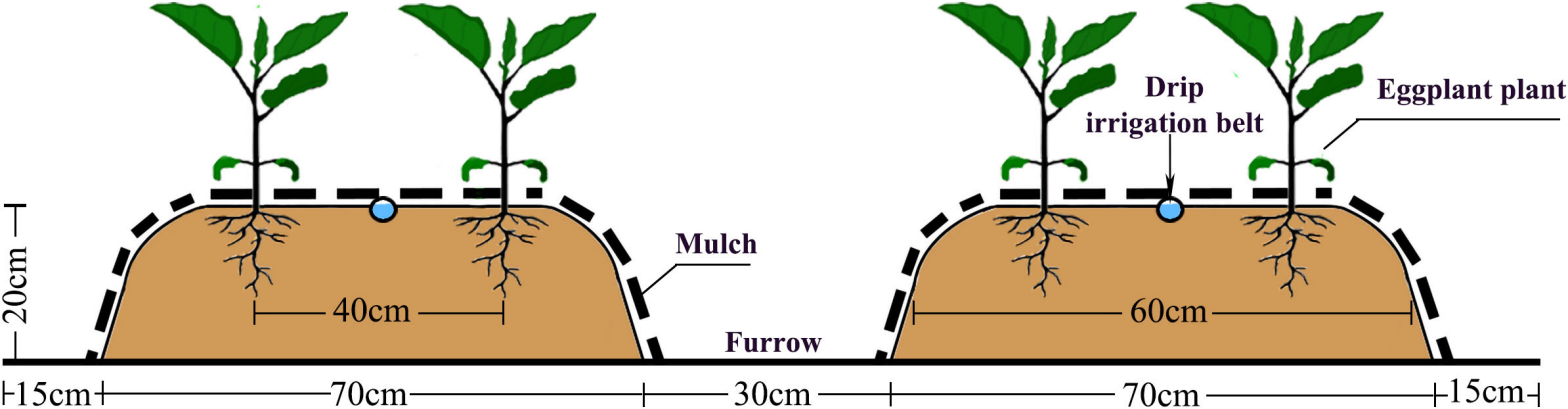
Schematic diagram of the eggplant planting layout in an experimental plot.

Eggplant ‘Lanza 2’ was used in the study. This cultivar has the advantages of high yield and strong resistance to disease. The eggplant seedlings were transplanted on May 9, 2021 and May 11, 2022. The plants were cultivated in an open field on ridges, each with two rows of plants, in combination with ridge mulching and mulched drip irrigation (**Figure 3**). Each plot comprised two ridges of length, width, and height of 600 cm, 60 cm, and 20 cm, respectively. A drip irrigation belt was laid along the center of each ridge, which was then covered with plastic film of 120 cm width of the film as a mulch. The discharge rate and drip hole distance of the drip irrigation belts were 2.4 L·h^−1^ and 30 cm, respectively. A gate valve with a pressure gauge and a water meter were installed on the branch pipe of each plot to adjust the water pressure and measure the water volume supplied. The eggplant seedlings were transplanted to both sides of the ridge, with spacing between rows and individual plants of 40 cm and 38 cm, respectively. An impervious film was buried vertically in the soil to 60 cm depth between each plot to prevent water infiltration. The soil moisture content was measured every 5–7 days for each treatment. When the soil moisture content was lower than the intended lower limit, it was irrigated to the intended upper limit.

Before transplanting, all plots received equal amounts of phosphate fertilizer (calcium superphosphate with 14% P_2_O_5_) and potassium fertilizer (potassium sulfate with 50% K_2_O), equivalent to 130 kg ha^−1^ P_2_O_5_ and 150 kg ha^−1^ K_2_O. Forty percent of the total nitrogen fertilizer (urea with 46% N) was applied before transplanting, and the remaining 60% nitrogen fertilizer was applied with irrigation at the flowering and fruit set, fruit development, and fruit ripening stages, respectively. All plots in the 2-year trial were regularly subjected to uniform agronomic management, such as timely weed removal, and pest and disease prevention. The fruit were harvested in four batches. The specific harvesting dates were July 15, August 1, August 15, and August 28 in 2021, and July 15, July 31, August 14, and August 30 in 2022. The entire growth period in each growing season comprised 112 days.

### 2.3 Measurements and calculations

#### 2.3.1 Soil moisture and crop evapotranspiration

Soil samples were collected at a location approximately 20 cm from the drip irrigation line in both growing seasons. The soil moisture content in the 0–100 cm soil layer was measured at 20 cm depth. Crop evapotranspiration (ET; mm) was calculated using the following equation (Yan et al., 2018; Li et al., 2022):


ET=P+I+U−D−R−ΔW

where *P*, *I*, *U*, *D*, *R*, and Δ*W* are the effective rainfall, irrigation volume, deep soil water supply to the tillage soil layer, deep seepage, surface runoff, and the variation in soil water storage within the 0–100 cm soil layer, respectively. The depth of the groundwater level at the experimental site was greater than 20 m, which could not be accessed by the crops, therefore *U* = 0. The experimental plots were relatively flat, watered by drip irrigation, and the intended soil moisture contents were lower than the FC; therefore, the surface runoff and deep seepage were essentially negligible, and thus were rated as *D* = 0 and *R* = 0. Hence, the formula for calculating crop evapotranspiration was adjusted to the following equation:


ET=P+I−ΔW

#### 2.3.2 Yield

The ripe fruit in each plot were harvested separately and the mean yield of the three replications per treatment was calculated.

#### 2.3.3 Water productivity and partial factor productivity of nitrogen

Water productivity (WP; kg m^−3^) is the yield of fruit harvested per unit volume of water consumed. Water productivity was calculated using the following equation (Pereira et al., 2012):


$$\text{WP}=\frac{Y}{\text{ET}}$$

where *Y* is the yield of eggplant (t ha^−1^).

The partial factor productivity of nitrogen (PFPn; kg kg^−1^) was calculated using the following equation (Li et al., 2017):


$$\text{PFPn}=\frac{Y}{\text{NFR}}$$

where NFR is the amount of nitrogen fertilizer applied (kg ha^−1^).

#### 2.3.4 Fruit quality

The soluble sugar content (SSC) was determined using the anthrone colorimetric method (Wang et al., 2011). Soluble protein (SP) content was quantified with the Komas Brilliant Blue method (Liu et al., 2019). Total soluble solids (TSS) content was measured using a digital pocket refractometer (PAL-1, ATAGO, Tokyo, Japan). Vitamin C (Vc) was detected using the 2,6-dichlorophenol indophenol sodium titration method (Liu et al., 2019).

#### 2.3.5 Calculation of optimal irrigation and nitrogen application regime for eggplant using TOPSIS

The optimal irrigation and nitrogen application regime that provided the best trade-offs in crop ET, yield, fruit quality, WP, and PFPn was calculated using the TOPSIS method, which comprises the following five steps (Luo and Li, 2018):

(1) Construct the original matrix:


$$X={\left(x_{ij}\right)}_{m\times n}=\left[\begin{array}{ccc} \begin{array}{cc} x_{11} & x_{12}\\ x_{21} & x_{22} \end{array} & \begin{array}{c} \ldots \\ \ldots \end{array} & \begin{array}{c} x_{1n}\\ x_{2n} \end{array}\\ \begin{array}{cc} \vdots & \vdots \end{array} & \ddots & \vdots \\ \begin{array}{cc} x_{m1} & x_{m2} \end{array} & \cdots & x_{mn} \end{array}\right]$$

where *x_ij_* (*i* = 1, 2…, *m*; *j* = 1, 2…, *n*) is the *j*th evaluation objective of the *i*th treatment.

(2) Normalize the original matrix:


$$Z_{ij}=W_{j}\frac{x_{ij}}{\sqrt{\sum_{i=1}^{m}x_{ij}^{2}}}$$

where *Z_ij_* is the standardization of *x_ij_*, and *w_j_* is the weight of the *j*th evaluation index. In this study, *w_j_* = 1.

(3) Determine the optimal solution *Z* ^+^ and the inferior solution *Z* ^−^:


$$Z^{+}=\left(Z_{max\;1},Z_{max\;2,}\cdots ,Z_{max\;n}\right)$$

$$Z^{-}=\left(Z_{min\;1},Z_{min\;2,}\cdots ,Z_{min\;n}\right)$$

(4) Calculate the distance *D_i_* ^+^ and *D_i_* ^−^ of each evaluation object from *Z* ^+^ and *Z* ^−^:


$$D_{i}^{+}=\sqrt{\sum_{j=1}^{n}{\left(Z_{ij}-Z_{j}^{+}\right)}^{2}}$$

$$D_{i}^{-}=\sqrt{\sum_{j=1}^{n}{\left(Z_{ij}-Z_{j}^{-}\right)}^{2}}$$

(5) Calculate the degree of proximity of each evaluation object to the optimal solution:


$$C_{i}=\frac{D_{i}^{-}}{D_{i}^{+}+D_{i}^{-}}$$

where 0< *C_i_* < 1; when *C_i_* approaches 1, the comprehensive evaluation effect will be approximately optimal.

### 2.4 Data analysis

Analysis of variance (ANOVA) was performed using IBM SPSS Statistics 23.0 software to analyze the data for water consumption, yield, WP, PFPn, and fruit quality. The significance of the differences between individuals means was assessed using Duncan’s multiple range test at the 95% significance level. Data processing and TOPSIS calculations were conducted using Microsoft Excel 2013 and Matlab 2017b. The figures were plotted using Origin 2021.

## 3 Results

### 3.1 Crop evapotranspiration

**Figure 4:**
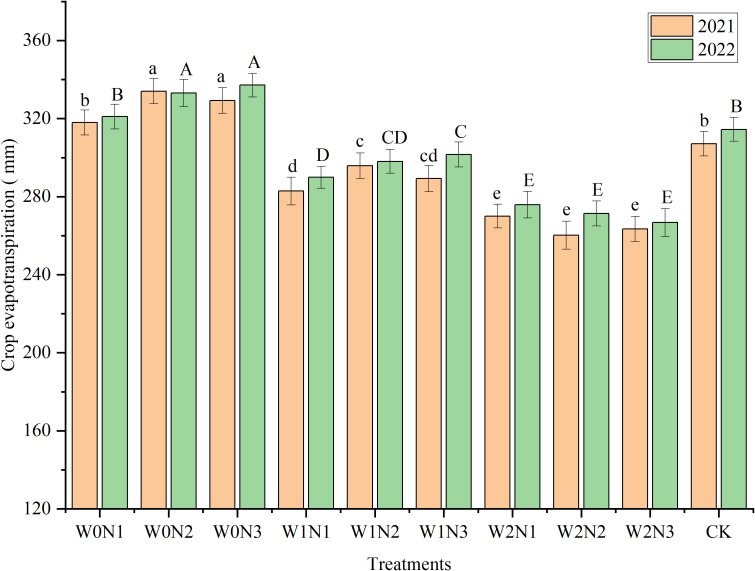
Effect of irrigation and nitrogen application treatments on the crop evapotranspiration (ET) of eggplant. Different lowercase letters or uppercase letters above bars within a year indicates a significant difference (*P*< 0.05).

The crop ET of all treatments in the 2-year experiment ranged from 260.23 mm to 337.19 mm (**Figure 4**). Irrigation had an extremely significant influence on ET (*P*< 0.001), whereas nitrogen application had a highly significant influence (*P*< 0.01) on ET (**Table 2**). The water × nitrogen interaction significantly influenced the ET of eggplant (*P*< 0.05). In 2021, the W0N2 treatment had the highest ET (334.23 mm), which was not statistically different from that of W0N3, whereas in the remaining treatments ET was significantly reduced by 4.81%–22.09% compared with that of W0N2 (*P*< 0.05). In 2022, the ET was highest in W0N3 (337.19 mm), which was not statistically different from that of W0N2, whereas the ET in the remaining treatments was significantly reduced by 4.79%–20.90% compared with that of W0N3 (*P*< 0.05). The ET of W1N2 in 2021 (295.85 mm) was 2.27% higher (*P* > 0.05) than that of W1N3 (289.27 mm). The ET in 2022 was slightly lower (*P* > 0.05) by 1.17% in W1N2 (298.07 mm) compared with that of W1N3 (301.61 mm). Compared with the CK, the ET of the W1N2 treatment was significantly reduced by 3.68% (2021) and 5.21% (2022). The mean ET was strongly reduced with increment in the degree of water deficit. At the N1, N2, and N3 nitrogen rates, no statistical differences in mean ET were observed.

**Table 2:** Effect of irrigation and nitrogen application treatments on crop evapotranspiration (ET), yield, crop water productivity (WP), and partial factor productivity of nitrogen (PFPn) of eggplant.

Variable	Year	W_0_	W_1_	W_2_	N_0_	N_1_	N_2_	N_3_	*F*_W_	*F*_N_	*F*_W×N_
ET (mm)	2021	322.09a	289.34b	264.55c	307.12a	290.29b	296.714b	293.97b	206.40***	8.35**	3.60*
	2022	326.44a	296.54b	271.33c	314.44a	295.61b	300.87b	301.84b	193.48***	6.35**	3.44*
Yield (t ha^−1^)	2021	73.76b	76.46a	61.24c	64.07c	67.63b	77.65a	69.38b	332.02***	136.42***	24.60***
	2022	75.68b	79.08a	62.84c	65.06c	69.48b	79.87a	71.79b	182.79***	75.43***	10.44***
WP (kg m^−3^)	2021	22.86b	26.41a	23.16b	20.87c	23.36b	26.15a	23.58b	37.35***	25.82***	3.14*
	2022	23.14b	26.65a	23.16b	20.69c	23.53b	26.52a	23.73b	112.98***	92.85***	8.77***
PFPn (kg kg^−1^)	2021	291.37a	289.61a	234.64b		314.57a	287.58b	213.47c	341.86***	900.07***	17.45***
	2022	299.82a	299.51a	240.55b		323.16a	295.83b	220.88c	163.87***	394.47***	5.50**

W and N are water deficit degree and nitrogen application rate, respectively. F_W_: the value of the split-plot ANOVA for water deficit degree; F_N_: the value of the split-plot ANOVA for nitrogen application rate; F_W×N_: the value of the split-plot ANOVA for interaction between water deficit degree and nitrogen application rate. * P< 0.05, ** P< 0.01, *** P< 0.001 (Duncan’s multiple range test). Different lowercase letters within a column indicates a significant difference (P< 0.05).

### 3.2 Plant yield

**Figure 5:**
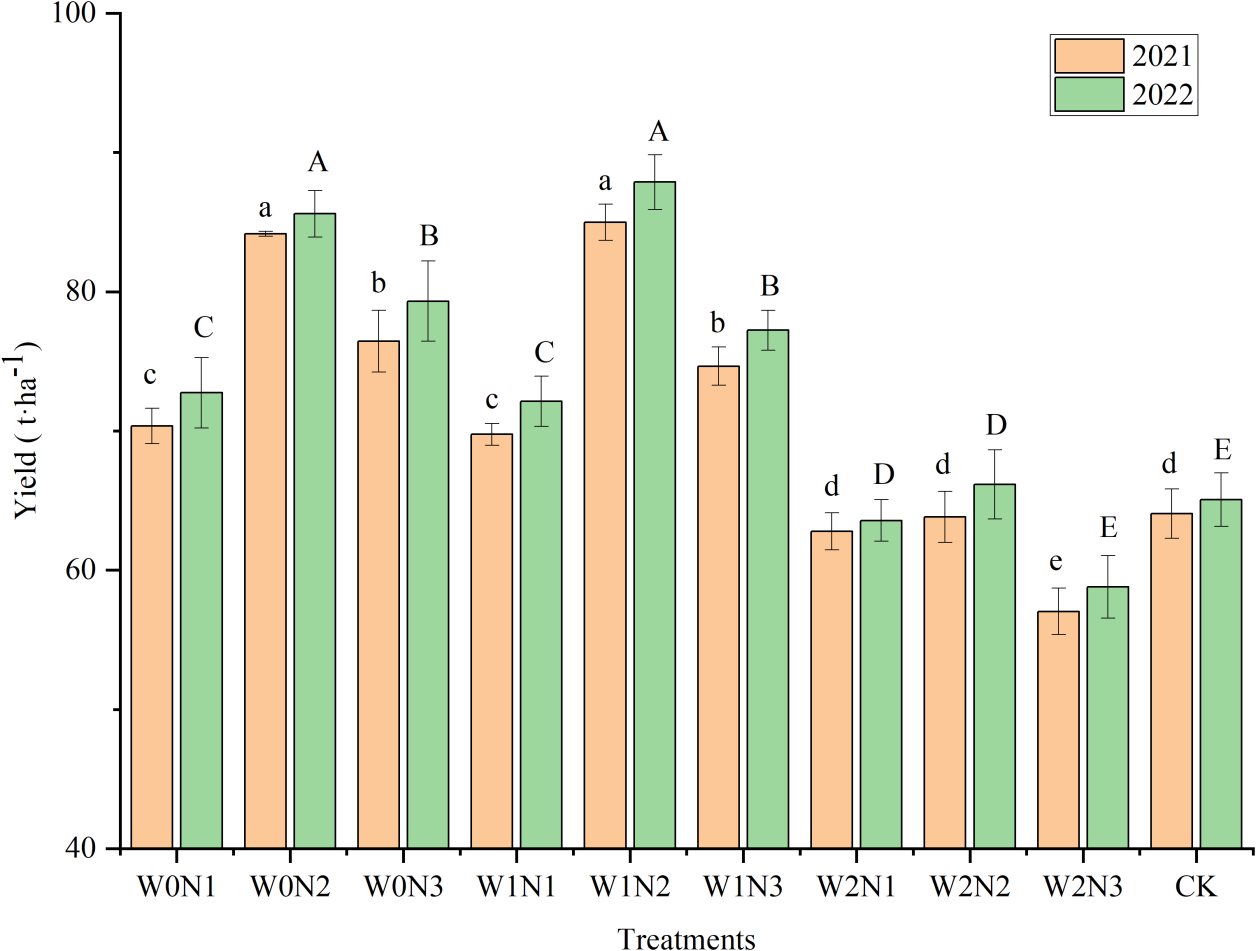
Effect of irrigation and nitrogen application treatments on yield of eggplant. Different lowercase letters or uppercase letters above bars within a year indicates a significant difference (*P*< 0.05).

Irrigation, nitrogen application, and water × nitrogen interaction had a highly significant (*P*< 0.001) influence on yield (**Table 2**). W1N2 had the highest yield, followed by W0N2, and no statistical difference between the yields of W1N2 and W0N2 was observed (**Figure 5**). The yields of W1N2 and W0N2 were significantly increased by 32.62% and 31.34% in 2021, and by 35.06% and 31.57% in 2022 compared with those of the CK (*P*< 0.05), respectively. The yield of W2N3 was the lowest among all treatments in both growing seasons (57.03 t ha^−1^ in 2021 and 58.81 t ha^−1^ in 2022), and was significantly lower by 10.99% and 9.61%, respectively, compared with that of the CK. The mean yield tended to increase and then decrease with increment in degree of water deficit. The highest yields were recorded at the W1 level (76.46 t ha^−1^ in 2021 and 79.08 t ha^−1^ in 2022) and the lowest at the W2 level (61.21 t ha^−1^ in 2021 and 62.84 t ha^−1^ in 2022). The yield at the W1 level was significantly higher by 3.66% (2021) and 4.49% (2022) compared with that of W0. The mean yield tended to increase and then decrease with increment in nitrogen rate. The maximum mean yield was attained at the N2 level, and was significantly higher than the yields at the other N levels. No statistical difference between the mean yield at the N1 and N3 levels was observed.

### 3.3 Water productivity and partial factor productivity of nitrogen

#### 3.3.1 Water productivity

**Figure 6:**
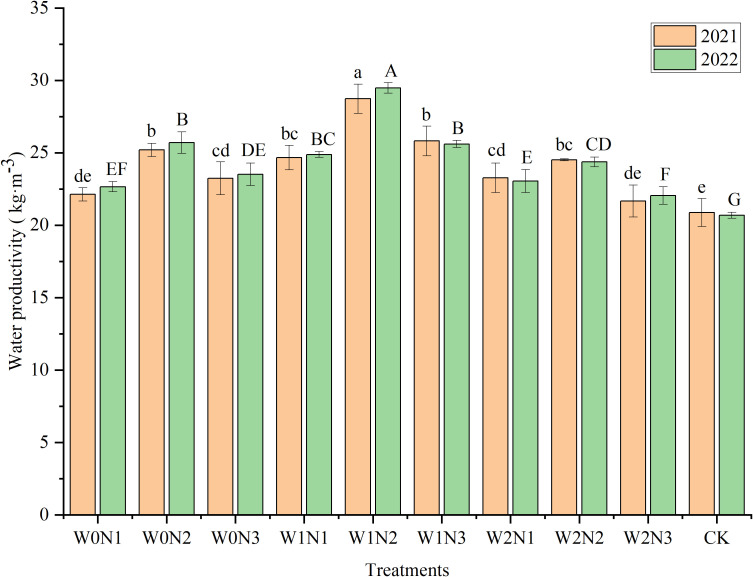
Effect of irrigation and nitrogen application treatments on water productivity (WP) of eggplant. Different lowercase letters or uppercase letters above bars within a year indicates a significant difference (*P*< 0.05).

Irrigation and nitrogen application showed a highly significant influence (*P*< 0.001) on WP in both 2021 and 2022 (**Table 2**). The interaction of irrigation and nitrogen application significantly influenced WP in 2021 (*P*< 0.05) and showed a highly significant influence on WP in 2022 (*P*< 0.001). The WP for each treatment ranged from 20.69 to 29.48 kg m^−3^ for both growing seasons (**Figure 6**). Among all treatments, WP was highest in W1N2 and lowest in the CK. The WP was dramatically higher in 2021 and 2022 by 37.84% and 42.48%, respectively, in W1N2 compared with that of the CK. At the same degree of water deficit, WP values tended to increase and then decrease with increment in nitrogen rate and the highest WP was observed at the N2 level. At the W0 level, the WP of N2 was significantly increased by 13.42% and 9.27% in 2022 compared with those of N1 and N3, respectively. At the W1 level, the WP of N2 was significantly higher by 18.49% and 15.11% in 2022 compared with N1 and N3, respectively. At the W2 level, the WP of N2 was significantly higher by 5.73% and 10.52% in 2022 compared with those of N1 and N3, respectively. The changes in WP in 2021 were similar to those observed in 2022. At the same rate of nitrogen application, WP followed a tendency to increase and then decrease with aggravation of water deficit. The mean WP did not differ dramatically between the W0 and W2 levels, whereas the WP at the W1 level was dramatically enhanced compared with those of the W0 and W2 levels. N2 had the highest mean WP. N1 and N3 showed no significant difference in mean WP, which was significantly lower by 10.67% and 9.83% in 2021, and by 11.27% and 10.52% in 2022, respectively, compared with that of N2.

#### 3.3.2 Partial factor productivity of nitrogen

**Figure 7:**
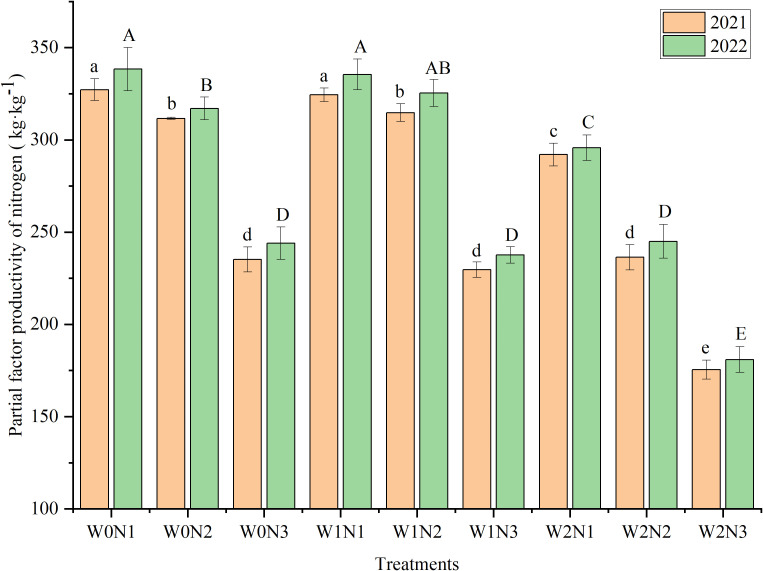
Effect of irrigation and nitrogen application treatments on partial factor productivity of nitrogen (PFPn) of eggplant. Different lowercase letters or uppercase letters above bars within a year indicates a significant difference (*P*< 0.05).

Irrigation, nitrogen application, and water × nitrogen interaction showed a highly significant influence (*P*< 0.001 or *P*< 0.01) on PFPn (**Table 2**). The treatment with the highest PFPn was W0N1, followed by W1N1, and no statistical difference was observed between the PFPn of W0N1 and W1N1 (**Figure 7**). The PFPn of W2N3 (175.49 kg kg^−1^ in 2021, 180.95 kg kg^−1^ in 2022) was significantly lower than those of the other treatments. At the same degree of water deficit, PFPn showed a gradual decreasing trend with increment in the nitrogen rate. The PFPn of the N1 and N3 levels declined dramatically with increase in degree of water deficit. In contrast, PFPn tended to increase and then decrease with increment in degree of water deficit at the N2 level. The mean PFPn decreased with the increase in degree of water deficit. No statistical difference in mean PFPn was detected between the W0 and W1 levels, whereas the mean PFPn at the W2 level was significantly lower by 19.47% (2021) and 19.77% (2022) compared with that of the W0 level, respectively. The mean PFPn declined dramatically with increment in the rate of nitrogen application. The mean PFPn at the N1 level was significantly higher by 47.36% and 46.31% in 2021 and 2022, respectively, compared with that of the N3 level.

### 3.4 Nutritional quality

#### 3.4.1 Soluble sugar content

**Figure 8:**
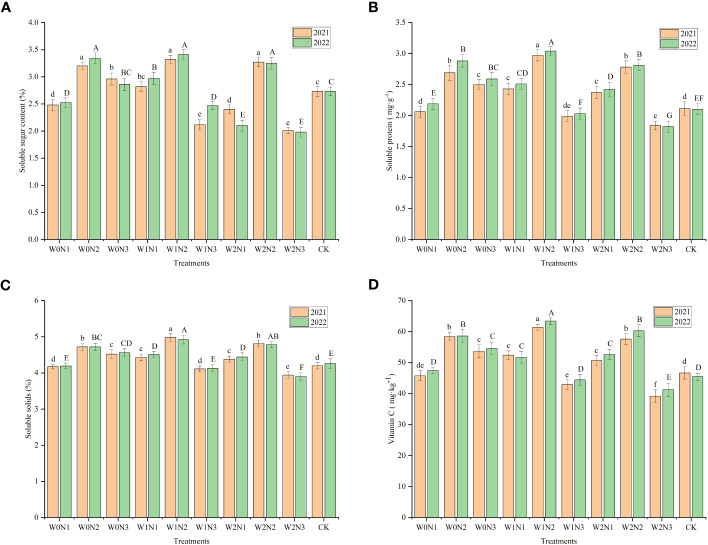
Effect of irrigation and nitrogen application treatments on fruit quality of eggplant. **(A–D)** Describes changes in soluble sugar, soluble protein, soluble solid, and Vc content for 2021 and 2022. Different lowercase letters or uppercase letters above bars within a year indicates a significant difference (*P*< 0.05).

The highest SSC was observed in the fruit of the W1N2 treatment (3.32 in 2021 and 3.41 in 2022), followed by W0N2 (3.20 in 2021 and 3.34 in 2022) and W2N2 (3.27 in 2021 and 3.25 in 2022); no statistical difference (*P* > 0.05) was observed among these three treatments, but all were significantly higher (*P*< 0.05) than the SSC of the other treatments (**Figure 8A**). The SSC of fruit treated with W1N2 was significantly increased by 21.61% (2021) and 24.91% (2022) compared with that of the CK. The W2N3 treatment had the lowest SSC (2.01 in 2021 and 1.98 in 2022) and was similar to that of W1N3. The SSC of W2N3 was significantly decreased by 26.37% (2021) and 27.47% (2022) compared with that of the CK. Irrigation, nitrogen application, and water × nitrogen interaction had a highly significant influence (*P*< 0.001) on fruit SSC (**Table 3**). The mean SSC decreased gradually with increase in degree of water deficit in 2021, but showed a tendency to rise and then decline with aggravation of water deficit degree in 2022. The mean SSC at the W0 level in 2021 was significantly higher by 3.27% compared with that of the W1 level (*P*< 0.05), whereas in 2022 the mean SSC did not differ significantly between the W0 and W1 levels. The mean SSC increased and then decreased with the increment in nitrogen rate.

**Table 3:** Effect of irrigation and nitrogen application treatments on the contents of mean soluble sugar (SSC), soluble protein (SP), total soluble solids (TSS), and vitamin C (Vc) of eggplant fruit.

Treatments	2021				2022			
	SSC (%)	SP (mg g^−1^)	TSS (%)	Vc (mg kg^−1^)	SSC (%)	SP (mg g^−1^)	TSS (%)	Vc (mg kg^−1^)
W_0_	2.84a	2.34b	4.40b	51.03a	2.86a	2.44b	4.43ab	51.47b
W_1_	2.75b	2.46a	4.51a	52.22a	2.95a	2.53a	4.52a	53.14a
W_2_	2.56c	2.33b	4.38b	49.14b	2.44b	2.35c	4.37b	51.38b
N_0_	2.74b	2.12c	4.20c	46.31c	2.73b	2.10c	4.26c	45.43c
N_1_	2.57c	2.29b	4.33b	49.60b	2.53c	2.38b	4.38b	50.55b
N_2_	3.26a	2.81a	4.84a	59.13a	3.33a	2.91a	4.81a	60.74a
N_3_	2.36d	2.11c	4.19c	45.24c	2.44c	2.15c	4.20c	46.72c
*F*_W_	33.19***	5.13*	5.27*	10.50**	80.74***	13.00***	5.50*	3.69*
*F*_N_	191.07***	103.51***	98.83***	107.67***	168.29***	128.88***	67.00***	116.47***
*F*_W*N_	51.62***	29.05***	22.35***	30.06***	25.93***	28.91***	20.85***	27.81***

W and N are water deficit degree and nitrogen application rate, respectively. F_W_: the value of the split-plot ANOVA for water deficit degree; F_N_: the value of the split-plot ANOVA for nitrogen application rate; F_W×N_: the value of the split-plot ANOVA for interaction between water deficit degree and nitrogen application rate. * P< 0.05, ** P< 0.01, *** P< 0.001 (Duncan’s multiple range test). Different lowercase letters within a column indicates a significant difference (P< 0.05).

#### 3.4.2 Soluble protein

Irrigation, nitrogen application, and water × nitrogen interaction had extremely significant influences on fruit SP content (*P*< 0.001), except that irrigation in 2021 had a significant effect (*P*< 0.05) on fruit SP content (**Table 3**). The SP content of W1N2-treated fruit was the highest (2.97 in 2021 and 3.04 in 2022) among the treatments, and was significantly increased (*P*< 0.05) by 40.76% (2021) and 44.76% (2022) compared with that of the CK (**Figure 8B**). The SP content was significantly reduced by 6.40%–38.05% (2021) and 5.26%–40.13% (2022) in all other treatments compared with that of W1N2. The W2N3 treatment had the lowest SP content, with a significant decrease of 12.80% (2021) and 13.33% (2022) compared with that of the CK. The mean SP content followed a tendency to increase and then decrease with aggravation of water deficit degree. The highest mean SP content was observed at the W1 level under mild water deficit in the two-year experiment, which was significantly higher by 3.56%–7.12% (*P*< 0.05) compared with that at the W0 and W2 levels. The mean SP content was significantly increased by 22.71% (2021) and 22.27% (2022) at the N2 level compared with N1 level, and by 33.18% (2021) and 35.35% (2022) at the N2 level compared with that of the N3 level. In addition, the mean SP content at the N1 level was significantly higher by 8.53% (2021) and 10.70% (2022) compared with that of the N3 level.

#### 3.4.3 Total soluble solids

The variation in TSS content in each treatment was similar to that observed for the SP content (**Figure 8C**). In the two growing seasons, irrigation significantly influenced the fruit TSS content (*P*< 0.05), whereas both nitrogen application and water × nitrogen interaction had a highly significant influence (*P*< 0.001) on TSS content (**Table 3**). The W1N2 treatment had the highest TSS content, which was significantly increased by 18.57% (2021) and 15.49% (2022) compared with that of the CK. The W2N3 had the lowest TSS content, which significantly decreased by 6.19% (2021) and 8.45% (2022) compared with the CK. Compared with W1N1 and W1N3, the TSS content in the W1N2 treatment was significantly increased. Compared with W1N2, the TSS content under the W0N2 treatment was significantly decreased by 5.22% (2021) and 4.07% (2022). Compared with W1N2, the TSS content in W2N2-treated fruit was significantly decreased by 3.41% in 2021 (*P*< 0.05). At the same water deficit degree, the TSS content were dramatically increased at the N2 level compared with the N1 and N3 levels. At the same nitrogen application level, the TSS content at the W0 and W2 levels was dramatically lower than that of the W1 level.

#### 3.4.4 Vitamin C

Both nitrogen application and water × nitrogen interaction had an extremely significant influence (*P*< 0.001) on fruit Vc content in the 2-year experiment (**Table 3**). Irrigation had a highly significant influence on fruit Vc content in 2021 (*P*< 0.01) and a significant influence on the Vc content in 2022 (P< 0.05). The highest Vc content was observed in the W1N2 treatment, which was significantly enhanced by 5.07%–43.12% (2021) and 5.21%–53.71% (2022) compared with the other treatments (**Figure 8D**). The Vc content of the W0N2 and W2N2 treatments was second only to that of W1N2, and no statistical difference was observed between W0N2 and W2N2. The Vc content in W2N3 was the lowest, which was significantly reduced by 16.03% (2021) and 9.22% (2022) compared with that of the CK. In 2021, the Vc content at the W0 and W1 levels was similar, whereas the mean Vc content at the W2 level was significantly decreased by 3.72% and 5.90% compared with those of the W0 and W1 levels (*P*< 0.05). However, in 2022, the Vc content at the W0 level was significantly reduced by 3.14% compared with that at the W1 level, and no statistical difference was observed between that at the W0 and W2 levels. The highest Vc content recorded was that of the N2 level, and at both the N1 and N3 levels the fruit had significantly lower Vc contents compared with that at the N2 level.

### 3.5 Trade-offs between ET, yield, WP, PFPn, and fruit quality

The optimum irrigation and nitrogen application treatments for eggplant were determined with consideration of the balance of ET, yield, water and nitrogen use efficiencies, and fruit quality. The ranking of TOPSIS scores for each treatment is shown in **Table 4**. The results for 2021 and 2022 were consistent, whereby the W1N2 treatment was ranked the optimum irrigation and nitrogen application treatments for eggplant, with *C_i_* values of 0.84 and 0.85 for 2021 and 2022, respectively. On average for both years, the W1N2 treatment resulted in the highest *C_i_* value (0.84) and provided the best overall benefits for balancing ET, yield, WP, PFPn, and quality in eggplant.

**Table 4:** Ranking of irrigation and nitrogen application management strategies for all treatments of eggplant calculated using the TOPSIS method.

Treatment	2021				2022				Mean value	
	*D* ^+^	*D* ^−^	*C_i_*	Rank	*D* ^+^	*D* ^−^	*C_i_*	Rank	*C_i_*	Rank
W0N1	0.72	0.41	0.36	8	0.70	0.42	0.38	8	0.37	8
W0N2	0.45	0.75	0.63	3	0.43	0.75	0.63	2	0.63	3
W0N3	0.58	0.53	0.48	5	0.61	0.51	0.46	5	0.47	5
W1N1	0.45	0.60	0.57	4	0.45	0.61	0.57	4	0.57	4
W1N2	0.18	0.94	0.84	1	0.17	0.94	0.85	1	0.84	1
W1N3	0.69	0.42	0.38	7	0.65	0.40	0.38	7	0.38	7
W2N1	0.59	0.51	0.47	6	0.64	0.50	0.44	6	0.45	6
W2N2	0.41	0.75	0.65	2	0.44	0.71	0.62	3	0.63	2
W2N3	0.93	0.36	0.28	9	0.93	0.38	0.29	9	0.28	9

D^+^ and D^−^ are positive and negative Euclidean distances, respectively; C_i_ is the comprehensive evaluation index; Rank is the ranking of the comprehensive evaluation scores.

## 4 Discussion

### 4.1 Effect of irrigation and nitrogen application treatments on plant yield

Water and nitrogen are the main limiting factors that affect crop yield (Mueller et al., 2012). Thus, irrigation and nitrogen application are critical to achieve higher crop yields (Du et al., 2021). This study showed that irrigation, nitrogen application, and water × nitrogen interaction had a highly significant influence on yield (*P*< 0.001). Some studies have indicated that reducing irrigation and nitrogen application in general may lead to a decrease in crop yield (Wang et al., 2017; Zhong et al., 2021). Nevertheless, in the present experiment, mild water deficit and a moderate nitrogen application level (W1N2) resulted in the highest fruit yield, which was significantly higher by 11.15% (2021) and 10.78% (2022) (*P*< 0.05) compared with that observed under adequate water supply and a higher nitrogen application level (W0N3). These results indicated that appropriate water deficit and nitrogen application were conducive to enhancement of eggplant yield, whereas excessive irrigation and nitrogen application were not only ineffective in improving eggplant yield, but also caused wastage of water and nitrogen fertilizer resources. This is consistent with previous findings that appropriate irrigation and nitrogen application are more beneficial than excessive application in water-scarce areas (Yang et al., 2017; Si et al., 2020). Rational irrigation and appropriate fertilization not only ensure optimal access to resources, but also enhance photosynthesis and carboxylation efficiency, thus significantly improving yield (Teixeira et al., 2014). The mean yield of eggplant under moderate water deficit (W2) was significantly decreased by 16.97% (2021) and 16.97% (2022) compared with that under adequate water supply (W0), and significantly decreased by 19.91% (2021) and 20.54% (2022) compared with that under mild water deficit (W1). The reduction of eggplant yield under moderate water deficit may be because nitrogen transport from the soil to the rhizosphere is adversely impacted by water deficit, thus inhibiting the effective use of nitrogen by plants (Kunrath et al., 2018). Under the same nitrogen application rate, the mean yield of eggplant first increased with increment in the nitrogen application level, and peaked under the N2 level, which may be because nitrogen application promotes the physiological growth of crops and is conducive for water and nutrient absorption by crops (Drenovsky et al., 2012). Nevertheless, eggplant yield decreased markedly when nitrogen application was raised from the N2 to the N3 level, and the lowest yield was observed in the W2N3 treatment with a significant decline of 10.99% (2021) and 9.61% (2022) compared with that of the CK (*P*< 0.05). This is because severe water deficit and a high rate of nitrogen application lead to increased osmotic pressure in the rhizosphere of plants, which inhibits transpiration and the nutrient absorption ability of plants, and ultimately results in decreased yield (Jalil Sheshbahreh et al., 2019).

### 4.2 Effect of irrigation and nitrogen application treatments on WP and PFPn

Enhancing water and nitrogen use efficiencies is especially valuable for sustainable agricultural development under water scarcity and excessive fertilizer application (Lu et al., 2021). The present irrigation and nitrogen applications had an extremely significant influence on WP content. Mild water deficit and medium nitrogen application (W1N2) resulted in the highest WP content, which was significantly higher by 11.27%–37.84% (2021) and 14.71%–42.48% (2022) compared with those of the other treatments. Mild water deficit reduced the ET of eggplant, while mild water deficit and a moderate nitrogen application rate resulted in an optimal combination of water and nutrients, resulting in higher eggplant yields and thus higher WP (Zhang et al., 2006). Furthermore, irrigation and nitrogen application by mulched drip irrigation enables precise control of the amounts of water and nitrogen applied, avoids evaporation and deep leaching of soil water as much as possible, and reduces nitrogen leaching (Lu et al., 2021). Kamran et al. (2023) found that WP under mild water deficit and mild nitrogen reduction was higher than that under other water and fertilizer treatments. The highest WP in winter wheat has been reported to be under water deficit and medium nitrogen application treatment (Lu et al., 2021). Suitable irrigation and nitrogen can simultaneously increase the availability of water and nutrients, promote the uptake and utilization of water and nutrients by crops, and thus improve WP and resource utilization (Dai et al., 2019). This effect of promoting complementarity is termed synergistic function (Wang et al., 2016). In this study, WP under the moderate water deficit (W2) treatment was not statistically different from that under the adequate water supply (W0) treatment, but was significantly reduced by 12.31% (2021) and 13.10% (2022) compared with that of the mild water deficit (W1) treatment. Given that the W2 treatment significantly reduced eggplant yield together with ET, and the reduction in yield was more pronounced than ET. The WP followed a tendency of a single-peaked curve with increment in nitrogen application rate. Under a high nitrogen rate (N3), the highest WP was under the W1N3 treatment, rather than the W0N3 treatment, because high nitrogen and adequate irrigation resulted in vigorous plant growth and increased the rate of unproductive transpiration (Li et al., 2021).

The PFPn is an indicator of nitrogen utilization (Li et al., 2021). Irrigation and nitrogen application based on crop demand are beneficial for improvement of resource utilization efficiency (Li et al., 2022). In this study, the PFPn under the high nitrogen application level (N3) was significantly reduced by 25.77% (2021) and 25.34% (2022) compared with that under the medium nitrogen application level (N2). This is because excessive nitrogen application exceeds the optimum requirement of eggplant, resulting in a decrease in nitrogen use efficiency, i.e. PFPn, which in turn causes nitrogen leaching and volatilization (Lu et al., 2021). In contrast, appropriate nitrogen application increases the PFPn and eggplant yield, while reducing soil nitrate loss (Spiertz, 2009). Optimum irrigation can improve crop nitrogen absorption, maximize nitrogen utilization efficiency, and enhance crop yield (Garnett et al., 2009). In contrast, excessive irrigation is detrimental to the improvement of nitrogen use efficiency (Li et al., 2017). Similar findings were observed in this study. The PFPn gradually declined with increase in the severity of water deficit, and no statistical difference in PFPn was detected under the adequate water supply (W0) and mild water deficit (W1) treatments, whereas the PFPn under moderate water deficit (W2) was significantly reduced compared with that under the other treatments. Mild water deficit and medium nitrogen application (W1N2) significantly increased PFPn compared with that under adequate water supply and high nitrogen application (W0N3). Moderate water deficit and nitrogen application increased PFPn in wheat compared with that under conventional irrigation and nitrogen treatments (Kamran et al., 2023). The PFPn of eggplant in the W2N1 treatment was significantly lower by 7.20% (2021) and 9.14% (2022) compared with that of the W1N2 treatment. This response was due to the severe water deficit and minimal nitrogen application rate, which failed to match the water and nutrient demands of the crop, resulting in insufficient biomass accumulation and yield reduction, thereby adversely affecting photosynthesis and reducing the nitrogen use efficiency (Tan et al., 2017; Si et al., 2020).

### 4.3 Effect of irrigation and nitrogen application treatments on fruit quality

The soil water content, which impacts soil nutrient transformation and nutrient uptake by plant roots, has a direct impact on fruit quality (Liu et al., 2019). Some studies have reported that water deficit dramatically improves the contents of SSC and TSS in fruit (Acevedo-Opazo et al., 2010; Ju et al., 2019; Yang et al., 2020). The present results revealed that mild water deficit greatly enhanced the contents of SSC, SP, and TSS in eggplant fruit. This is similar to the results of Yang et al. (2019), who observed that mild drought enhances fruit quality parameters compared with those under adequate water supply. Under water stress, the phloem sap flow to the fruit is hindered, resulting in an increase in the solute concentration of the sap and decrease in water transport from the xylem to the fruit (Guichard et al., 2001). However, the decrease in fruit water content barely affects the accumulation of sugars, leading to an enhancement in sugar concentration while improving fruit quality (Chen et al., 2014). In addition, water deficit favors the storage of starch and the conversion of starch to sugar, which increase the TSS and SSC contents (Wang et al., 2011). Vitamin C, an additional indicator of the nutritional quality of fruit, participates in various metabolic reactions in the human body (Lee and Kader, 2000). The present study revealed that mild water deficit in 2022 strongly improved the Vc content in eggplant fruit. This result is consistent with previous findings that water stress substantially enhances the accumulation of Vc (Liu et al., 2019). This may be because water stress diminishes the leaf area of plants, but strengthens the light intensity and time in the canopy, which promotes the synthesis of Vc (Dumas et al., 2003).

The contents of SSC, organic acids, TSS, and Vc in tomato fruit are significantly enhanced when the nitrogen application rate does not exceed 300 kg ha^−1^ (Li et al., 2021). This research found that the contents of SSC, SP, TSS, and Vc were significantly increased in fruit under a medium nitrogen application rate (N2). This is explained by the observation that a rational nitrogen level is beneficial to nitrogen absorption by plants, and thus improves photosynthetic activity and protein synthesis (Wang et al., 2021; Nasar et al., 2022). Nevertheless, under a high nitrogen application level (N3), the contents of SSC, SP, TSS, and Vc in eggplant fruit were decreased significantly. Excessive nitrogen application constrains nutrient transport to the fruit, but enhances the synthesis of amino acids and proteins in organic acids, thus increasing sugar consumption and reducing sugar accumulation in the fruit (Sun et al., 2011). In addition, excessive nitrogen application facilitates the vegetative growth of crops, and the rapid increase in leaf area leads to expansion of the shaded area and reduction in temperature, which ultimately induces acid synthesis and is not beneficial to the accumulation of SSC (Benard et al., 2009). However, Vc synthesis requires the participation of sugars, and reduction in the SSC inhibits Vc synthesis (Wang et al., 2017).

### 4.4 Determination of optimum deficit irrigation and nitrogen management strategies

The optimum deficit irrigation and nitrogen management strategies should consider ET, yield, WP, PFPn, and fruit quality. In the current study, adequate water supply (W0) increased PFPn, but decreased yield, WP, and fruit quality of eggplant. At the medium nitrogen application level (N2), the ET during the growth period was reduced and PFPn was significantly decreased, whereas the yield, fruit quality, and WP were improved. Given that the impacts of irrigation and nitrogen application management on yield, WP, PFPn, and fruit quality of eggplant involve complex interaction effects, an optimal balance between these indicators cannot be determined by qualitative analysis alone. Therefore, the quantitative relationships among these indicators were assessed with the TOPSIS method. The TOPSIS results showed that the W1N2 treatment ranked first in the 2-year trial. Consequently, the combination of W1 (60%–70% FC) and N2 (270 kg ha^−1^) provided the optimal response (**Table 4**), and was the best irrigation and nitrogen application management option for cultivated eggplant at the study site.

## 5 Conclusion

Mild water deficit significantly increased the yield, WP, and SP and Vc contents of eggplant, but slightly decreased PFPn and significantly decreased the crop ET. A high nitrogen application rate significantly reduced the yield, WP, PFPn, and fruit quality, but had no significant effect on ET. A comprehensive evaluation using TOPSIS indicated that mild water deficit and a medium nitrogen application level (i.e., W1N2) had the best comprehensive effect and was the best irrigation and nitrogen application management strategy to balance ET, yield, WP, PFPn, and fruit quality of eggplant in a cold and arid environment. The present findings contribute novel insights and a theoretical basis for water and nitrogen management of eggplant in a cold and arid environment. However, climatic variables (such as rainfall) and soil conditions vary in different regions. Therefore, the mechanisms by which climate change and soil conditions in different regions influence the effects of deficit irrigation and nitrogen application on the yield, fruit quality, and resource utilization of eggplant require further investigation.

## Data availability statement

The original contributions presented in the study are included in the article/supplementary material. Further inquiries can be directed to the corresponding author.

## Author contributions

CZ prepared the experimental scheme, data analysis and drafted the article. HZ was responsible for the funding acquisition. HZ and SY revised the experimental protocol and article format. CZ, XC, FL, YW, YYW and LL performed part of the experiments and provided some of the experimental results for the manuscript. All authors contributed to the article and approved the submitted version.
